# Why is Aged Acetylcholinesterase So Difficult to Reactivate?

**DOI:** 10.3390/molecules22091464

**Published:** 2017-09-04

**Authors:** Daniel M. Quinn, Joseph Topczewski, Nilanthi Yasapala, Alexander Lodge

**Affiliations:** Department of Chemistry, University of Iowa, Iowa City, IA 52242, USA; jtopczew@umn.edu (J.T.); nilanthi.yasapala@yahoo.com (N.Y.); ALodge@foley.com (A.L.)

**Keywords:** acetylcholinesterase, organophosphorus agent, inhibition, reactivation, aging, transition state

## Abstract

Organophosphorus agents are potent inhibitors of acetylcholinesterase. Inhibition involves successive chemical events. The first is phosphylation of the active site serine to produce a neutral adduct, which is a close structural analog of the acylation transition state. This adduct is unreactive toward spontaneous hydrolysis, but in many cases can be reactivated by nucleophilic medicinal agents, such as oximes. However, the initial phosphylation reaction may be followed by a dealkylation reaction of the incipient adduct. This reaction is called aging and produces an anionic phosphyl adduct with acetylcholinesterase that is refractory to reactivation. This review considers why the anionic aged adduct is unreactive toward nucleophiles. An alternate approach is to realkylate the aged adduct, which would render the adduct reactivatable with oxime nucleophiles. However, this approach confronts a considerable—and perhaps intractable—challenge: the aged adduct is a close analog of the deacylation transition state. Consequently, the evolutionary mechanisms that have led to transition state stabilization in acetylcholinesterase catalysis are discussed herein, as are the challenges that they present to reactivation of aged acetylcholinesterase.

## 1. Introduction

**Figure 1:**
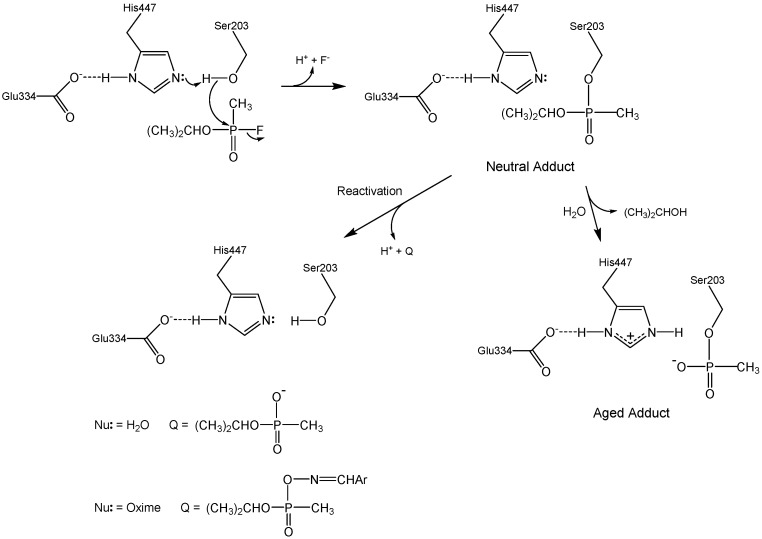
Inhibition of AChE by the organophosphorus nerve agent sarin.

Figure 1 outlines the various chemical reactions that ensue when acetylcholinesterase (AChE) is exposed to organophosphorus (OP) inhibitors. These reactions are of considerable medicinal and national security interest, since OP agents that inhibit AChE (such as sarin in Figure 1) are acute neurotoxins. Events in recent years underscore this concern: Iraq used tabun against Iranian troops in the Iran–Iraq war [1], terrorists have used sarin against civilians [2], tyrants have used sarin to mass murder their own people [3], and a dictator ordered the execution of his own brother with VX [4].

The seminal chemical reaction between sarin and AChE in Figure 1 is attack of the active site serine at phosphorus with concomitant displacement of the fluoride leaving group. This initial neutral adduct is an analog of the transition states in the acylation stage of AChE catalysis [5], and its production is accelerated by the catalytic machinery of the active site; i.e., the Ser-His-Glu catalytic triad, the oxyanion hole, and the acyl binding site [6,7]. The initial adduct is trenchantly unreactive toward spontaneous hydrolysis (cf. Figure 1, Nu: = H_2_O), a reaction that therefore is of no medicinal import. The lack of hydrolytic reactivity of the initial adduct is rationalized herein in terms of the resemblance of the adduct to the transition states of the acylation stage of AChE catalysis. However, judiciously designed oxime nucleophiles can readily dephosphylate the initial adducts, as shown in Figure 1 (Nu: = ArCH=NOH). 2-Pyridinealdoxime methiodide (2-PAM) is a component of the FDA approved standard countermeasure that is in use in the United States [8]. Unfortunately, the initial neutral adduct may convert to a monoanionic aged adduct via a dealkylation reaction, as shown in Figure 1. This aging reaction is accelerated by cation-π interaction between Trp86 of the active site and the alkyl fragment that departs in the carbocationic transition state [9]. The aged adduct is remarkably unreactive. Despite considerable efforts over the last two generations, no practicable means for reactivating the aged adduct have been found, and therefore no medicinal agents are available for aged AChE. Why is aged AChE so difficult to reactivate? The answers to this question are framed in the following passages in terms of the structural and energetic features of the aged AChE adduct, and will hopefully inform future efforts to solve the knotty problem that reactivation of aged AChE poses.

## 2. Discussion

**Figure 2:**
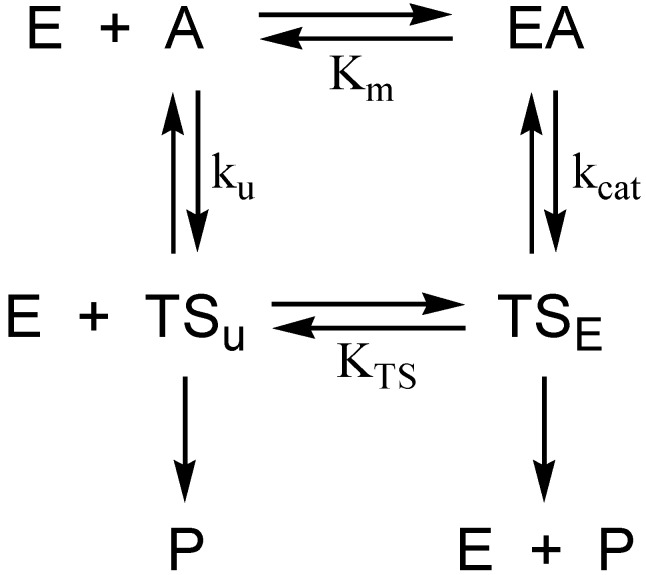
Thermodynamic cycle for estimation of transition state stabilization in AChE catalysis. E, EA, A, P, TS_u_, and TS_E_ are respectively free enzyme, Michaelis complex, substrate, product, transition state of the spontaneous hydrolysis reaction, and transition state of the AChE-catalyzed reaction. K_m_ and k_cat_ are the respective Michaelis constant and turnover number of the AChE-catalyzed reaction; k_u_ and K_TS_ are respectively the rate constant of the spontaneous hydrolysis reaction and the dissociation constant of the enzymic transition state.

Consideration of the selective pressure that guided the evolution of the catalytic power of AChE provides a framework for understanding the hydrolytic stability of the initial phosphyl adduct and the remarkable unreactivity of aged AChE. The physiological context in which AChE operates is cholinergic neurotransmission in the central and peripheral nervous systems. Since cholinergic neurotransmission occurs on a millisecond to second time scale, it is apparent that the evolution of the catalytic power of AChE has been beset with the “need for speed”. Indeed, AChE is among the most potent of biocatalysts that accelerate hydrolysis reactions. The second-order rate constant k_cat_/K_m_ exceeds 10^9^ M^−1^·s^−1^ at low ionic strength [10], while the turnover number k_cat_ > 10^4^ s^−1^ [11]. For k_cat_/K_m_, the rate constant is prominently diffusion controlled; i.e., the enzyme is functioning at the “speed limit” of biological catalysis [12]. A yet more telling analysis arises from consideration of rate constants in the thermodynamic cycle of Figure 2. The cycle is numerically informed by the catalytic constants of AChE catalysis, and by Wolfenden’s determination of the rate constant for nonenzymic neutral hydrolysis of acetylcholine, k_un_ = 7.2 × 10^−9^ s^−1^ [13]. These values allow one to calculate the catalytic acceleration effected by the enzyme when substrate concentration is << K_m_, a ratio that Wolfenden calls the catalytic proficiency of the enzyme [14]:

(1) Catalytic Proficiency = kcatKmkun= 1.4 × 1017 M−1

The reciprocal of the catalytic proficiency is the thermodynamic dissociation constant of the acylation transition state, K_TS_, from which one can calculate the free energy of dissociation of the acylation transition state at T = 298 K, as in Equation (2):

(2) $${\Delta G}_{\mathrm{TS}}=\;-{\mathrm{RTlnK}}_{\mathrm{TS}}=98\;\mathrm{kJ}/\mathrm{mol}\;\left(23\;\mathrm{kcal}/\mathrm{mol}\right)$$

This analysis shows that, for AChE, and indeed for any enzyme catalyzed reaction, catalytic power must derive from transition state stabilization. For AChE, the acylation transition state is 98 kJ/mol more stable than the transition state of the spontaneous hydrolysis of acetylcholine, an observation that can be interpreted in terms of the elements of molecular recognition that the enzyme brings to bear on the acylation transition state [6]. A similar analysis can be considered when AChE operates under conditions of substrate saturation; i.e., k_cat_ is rate limiting:

(3) $$Catalytic\;Acceleration\;=\;\frac{k_{\mathrm{cat}}}{k_{\mathrm{un}}}=\;1.4\;\times \;10^{12}$$

Since it is likely that k_cat_ is rate limited by the deacylation stage of catalysis [15], one can calculate the stabilization of the deacylation transition state as −RTln(1.4 × 10^12^) = −69 kJ/mol (−17 kcal/mol).

How is the notable transition state stabilization that AChE effects related to inhibition by OP agents? This is a question that was posed by Ashani and Green in 1982 [16], and that is approached herein from structural and energetic perspectives. The analysis that follows is informed by the availability in the literature of crystal structures of both aged and neutral phosphyl-AChE adducts [17,18,19,20]. Consider the acylation transition state for AChE catalyzed hydrolysis of acetylthiocholine (ATCh). By measuring β-deuterium secondary isotope effects on k_cat_/K_m_, Quinn and collaborators showed that the bond order between the γO of Ser203 of human AChE and the carbonyl carbon of ATCh is 0.8 ± 0.2 in the acylation transition state [5]. Moreover, the π-bond of the ATCh carbonyl function is extensively broken. These respective bonds have bond lengths of 1.45 Å and 1.38 Å, as was found in a simple computational model of the acylation transition state [5]. The corresponding distances in the neutral phosphyl adduct that results when *T. californica* AChE is inhibited by VX are 1.57 Å and 1.47 Å. The similarity among angles about the erstwhile carbonyl carbon of the transition state model and the phosphorus of the neutral VX adduct are yet more remarkable. For example, in the transition state model, the bond angle for nucleophile oxygen to carbonyl carbon to carbonyl oxygen is 106.6°, while in the VX adduct the corresponding angle is 109.5°; in the transition state model the acyl methyl to carbonyl carbon to carbonyl oxygen angle is 113.0°, while in the VX adduct the corresponding angle is 114.7°. The notable similarity between transition state structural features and those of the initial phosphyl adduct suggests that the phosphyl adduct is trenchantly unreactive toward spontaneous hydrolysis, because AChE stabilizes the adduct in much the same way that it stabilizes the acylation transition state. 

A comparable analysis can be considered for the aged enzyme. Though a detailed structure of the deacylation transition state is not available, Quinn and coworkers have shown by measurements of β-deuterium secondary isotope effects on k_cat_ that for the deacylation stage of cholinesterase catalysis the Michaelis complex that accumulates on the enzyme in the steady state is a tetrahedral intermediate [21,22,23]. In nonenzymic ester hydrolysis the tetrahedral intermediate is at least 11 kcal/mol (46 kJ/mol) less stable than the ester from which it comes [23]. Therefore, the tetrahedral intermediate in the deacylation stage of AChE catalysis is stabilized by at least 46 kJ/mol (11 kcal/mol). This analysis shows that the stabilization of the tetrahedral intermediate is a large fraction of the 69 kJ/mol stabilization of the deacylation transition state that was calculated from the thermodynamic cycle of Figure 1. Consequently, it is unsurprising that, despite two generations of effort by medicinal chemists, a nucleophilic antidote for aged AChE has yet to be found. The trenchant unreactivity of aged AChE arises because the aged adduct is a close structural analog of the tetrahedral intermediate in the deacylation stage of catalysis, and of the transition states for formation and decomposition of the intermediate.

**Figure 3:**
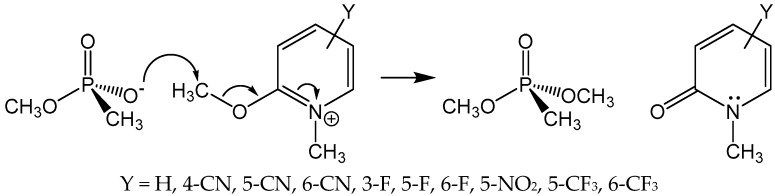
Substituted 2-Methoxy-1-methylpyridiniums as phosphonate anion methylating agents. Reactions were run in DMSO-d_6_ at 25 °C and were monitored by ^1^H-NMR spectroscopy [24].

A seemingly obvious approach to reactivate aged AChE is to synthesize and evaluate putative medicinal agents that can realkylate the monoanionic aged adduct, which would produce anew a neutral phosphyl adduct that can be reactivated by nucleophilic medicinal reagents, such as 2-PAM. Accordingly, Topczewski and Quinn [24] reported that various substituted 2-methoxy-1-methylpyridiniums were reactive as methyl transfer agents to the methoxyl methyphosphonate anion in the reaction shown in Figure 3. This reaction was chosen as an analog of methyl transfer to the aged AChE adduct. The 2-methoxy-1-methylpyridinium agents alkylated the phosphonate anion with a range of rates that was described by a multiple linear free energy relationship, with the most reactive agent (Y = 3-F in Figure 3) effecting 40% methyl transfer in 10 min. Despite these very promising model reaction results, none of the 2-methoxy-1-methylpyridinium reagents gave a 2-PAM reactivatable phosphyl adduct with aged human AChE.

The secret behind the unreactivity of aged AChE toward methylation with 2-methoxy-1-methylpyridinium reagents again surely lies in the fact that the aged adduct is a close structural analog of the deacylation transition state. Consider the following analysis. The p*K*_a_ of the conjugate acid of methoxyl methylphosphonate anion, measured by ^31^P-NMR spectroscopy, is 1.9 (Topczewski, J.J.; Quinn, D.M., unpublished observation). As Harel et al. [6] discussed, the contribution of the oxyanion hole to transition state stabilization in AChE catalysis is at least 21 kJ/mol (5 kcal/mol). One can reasonably expect that this stabilization will result in a comparable stabilization of the anionic phosphyl adduct of aged AChE, which would lower the conjugate acid p*K*_a_ of the aged adduct by 4 pK units. The decreased p*K*_a_ of the phosphyl adduct should in turn decrease the reactivity of the adduct as a methyl transfer nucleophile to an extent that is accommodated by a Brönsted relationship [25]:

(4) $$\frac{k_{\mathrm{nnc}}}{k_{0}}=\;10^{\beta_{\mathrm{nuc}}\Delta pK_{a}}$$

In this equation, k_0_ is the rate constant for methyl transfer measured in the model reaction of Figure 3, k_nuc_ is the expected rate constant for aged AChE, Δp*K*_a_ = −4 as discussed above, and β is a measure of the degree of methyl transfer that has been achieved in the transition state of the methyl transfer reaction. Since methyl transfer is between oxyanions, it is reasonable to estimate that β = 0.5. Consequently, in aged AChE, methyl transfer will occur at least 100-fold more slowly than in the model reaction. For the most reactive of the 2-methoxy-1-methypyridiniums of Figure 3, which had a half-life of 20 min, this analysis predicts that in aged AChE the half-life will be at least 2000 min = 33 h, which is much too slow to be of use in a therapeutic context. It should be noted that the aqueous p*K*_a_ of methoxyl methanephosphonate has been used in this analysis for reactivity in the model reaction in DMSO. It is well known that conjugate acid p*K*_a_s of oxyanions are elevated in DMSO versus aqueous solution, and therefore this analysis certainly underestimates the unreactivity of aged AChE. Nonetheless, the general conclusion remains. Because the aged adduct is a close structural mimic of the tetrahedral intermediate in the deacylation stage of catalysis, and of the transition states leading to and from the intermediate, the intrinsic nucleophilic reactivity of the aged enzyme is compromised by the elements of molecular recognition that have evolved as the catalytic power of AChE has evolved.

## 3. Conclusions

The analysis discussed herein suggests that intrinsic unreactivity is a major factor in the inability of medicinal chemists to find an antidote to aged AChE over the last 60 years. The challenge that this problem presents is considerable. As discussed herein, the unreactivity of aged AChE is a result of the evolution of the catalytic power of the enzyme itself. However, there is reason to be optimistic that a solution to this conundrum will be found. An integrated effort that involves medicinal chemistry, biochemistry, and computational chemistry may well reveal additional elements of molecular recognition, reactivator design, and reactivator mechanism that experimenters can harness to produce agents that can covalently modify aged AChE, albeit with sufficient reactivity to be useful in a therapeutic context.
